# A matter of taste: the adverse effect of pollen compounds on the pre-ingestive gustatory experience of sugar solutions for honeybees

**DOI:** 10.1007/s00359-019-01347-z

**Published:** 2019-06-05

**Authors:** E. Nicholls, S. Krishna, O. Wright, D. Stabler, A. Krefft, H. Somanathan, N. Hempel de Ibarra

**Affiliations:** 1 Centre for Research in Animal Behaviour, University of Exeter, Exeter, UK; 2 Present Address: School of Life Sciences, University of Sussex, Brighton, UK; 3 Centre for Research in Ecology and Evolution, Indian Institute of Science Education and Research Thiruvananthapuram (IISER-TVM), Thiruvananthapuram, India; 4 Institute of Neuroscience, University of Newcastle, Newcastle, UK

**Keywords:** Honeybee, Insect behaviour, PER, Olfactory learning, Foraging

## Abstract

In addition to sugars, nectar contains multiple nutrient compounds in varying concentrations, yet little is known of their effect on the reward properties of nectar and the resulting implications for insect behaviour. We examined the pre-ingestive responses of honeybees to sucrose solutions containing a mix of pollen compounds, the amino acids proline or phenylalanine, or known distasteful substances, quinine and salt. We predicted that in taste and learning assays, bees would respond positively to the presence of nutrient compounds in a sucrose solution. However, bees’ proboscis extension responses decreased when their antennae were stimulated with pollen- or amino acid-supplemented sucrose solutions. Compared to pure sucrose, bees exhibited worse acquisition when conditioned to an odour with pollen-supplemented sucrose as the unconditioned stimulus. Such learning impairment was also observed with quinine-containing sucrose solutions. Our results suggest that bees can use their antennae to detect pollen compounds in floral nectars. Depending on the type and concentrations of compounds present, this may result in nectar being perceived as distasteful by bees, making it less effective in reinforcing the learning of floral cues. Such reward devaluation might be adaptive in cases where plants benefit from regulating the frequency of bee visitation.

**Electronic supplementary material:**

The online version of this article (10.1007/s00359-019-01347-z) contains supplementary material, which is available to authorized users.

## Introduction

Bees are capable of assessing the value of nectar rewards offered by flowers on the basis of sugar concentration and volume (Núñez 1970), but relatively little is known as to how other compounds may affect the taste of nectar for pollinators, and the subsequent effects on behaviour. This question is of importance, given that nectar is more than just sugar water and frequently contains a wide variety of other compounds, of which amino acids are the most abundant (Baker and Baker 1973, 1975, 1977; Gottsberger et al. 1984; Nepi 2014). Since learning plays an important role in shaping the foraging decisions of bees (Menzel 1985), the taste of nectar may have a direct impact on the number of pollinator visits a flower receives (Gardener and Gillman 2002; Petanidou et al. 2006; Stevenson et al. 2017).

Variation in the composition and concentration of compounds present in nectar is high, both within and across flower species (Gottsberger et al. 1989; Gardener and Gillman 2001; Power et al. 2018). For example, when testing the concentration of free amino acids detected in the nectar of eight plant species, Gottsberger et al. (1989) observed a 13-fold difference between the species with the highest median concentration of nectar amino acids (*Hibiscus rosa*-*sinensis*, 254 µg/ml) and the lowest (*Abutilon pictum cv. Thompsonii,* 19 µg/ml). In addition to compounds directly secreted into nectar by the plant itself, recent work has shown that bacterial activity may also change the composition of amino acids in nectar and impact plant–pollinator relationships (Herrera et al. 2009; Vannette et al. 2013). Contamination of nectar with pollen grains, dislodged during flower visits by insects, can also result in a notable increase in amino acid concentration (Gottsberger et al. 1990; Erhardt and Baker 1990).

Within a certain range of concentrations, honeybees have been shown to prefer feeding on nectars or artificial sugar solutions containing amino acids over those containing sugars alone (Inouye and Waller 1984; Alm et al. 1990; Kim and Smith 2000; Carter et al. 2006; Petanidou et al. 2006; Hendriksma et al. 2014), leading to the suggestion that amino acids may serve as an additional nutritional reward for visiting pollinators (Baker and Baker 1973, 1975, 1986; Gottsberger et al. 1984; Pacini and Nepi 2007; Rodríguez-Peña et al. 2013). However, at high concentrations, for example, within the 100 mM range for proline and phenylalanine (Simcock et al. 2014), any positive effect on feeding tends to dissipate (Inouye and Waller 1984; Carter et al. 2006; Simcock et al. 2014), and certain amino acids, such as serine, are not at all attractive to bees (Bertazzini et al. 2010). This suggests that food intake decisions during nectar assessment by bees are influenced by a balance of appetitive and aversive responses. Such decision involve both pre- and post-ingestive sensory cues of differences in reward composition; however, the gustatory mechanisms are still little understood (for a review see Wright et al. 2018).

Here, we focus on the antennae as pre-ingestive taste organs, to address whether bees can taste differences between sugar solutions containing different additional compounds and if the presence of such compounds impacts on their learning performance. Like other insects, bees have gustatory receptors located across their body, for example in their brain and guts; however, the highest concentration and expression levels of the twelve gustatory receptor genes are in the mouth parts and antennae (Robertson and Wanner 2006; Simcock et al. 2017). The antennae are a multimodal organ which bees actively move to touch surfaces, for example when landing on a flower or searching for food (Ribbands 1949; Kisch and Erber 1999; Haupt 2004; Lunau et al. 2009; Evangelista et al. 2010). When the antennae of a motivated forager or hungry bee make contact with a sugar solution, the feeding response is initiated by extension of the proboscis. The decision of whether to extend the proboscis, when to retract it, and how much to imbibe, is dependent on both the quality of the reward and the bee’s internal or nutritional state and expectations (Núñez 1966; Varju and Núñez 1993; Gil et al. 2008; Simcock et al. 2014), with limits set by the suction mechanics of their mouthparts and the capacity of the gut organs (Blatt and Roces 2001; Kim et al. 2011). How pre-ingestive perception of reward quality via the antennae contributes to phago-stimulatory and phago-inhibitory control of the feeding response in pollinating insects is  still unknown for floral rewards that contain additional non-sugar compounds.

It is well established that perception of reward quality influences how bees learn (e.g. Bitterman et al. 1983; Loo and Bitterman 1992; Menzel and Müller 1996; Scheiner et al. 2005). For instance, experiments with harnessed bees show that more concentrated sugar solutions lead to faster learning of conditioned odours in honeybees (Bitterman et al. 1983). There is evidence to suggest that supplementation of sugar solutions with particular amino acids can also improve learning. Kim and Smith (2000) found that glycine, a common component of both nectar and pollen, leads to an enhanced conditioned response and resistance to extinction, relative to a group receiving sucrose alone as the unconditioned stimulus (US). Wright et al. (2009) also demonstrated, via differential reinforcement, that supplementing the sucrose US with proline leads to an improvement in learning. Both results suggest that amino acids serve to enhance the value of the sucrose reward, leading to more robust acquisition of the conditioned response.

The proboscis extension response (PER) paradigm, widely used to study learning in honeybees, typically involves pre- and post-ingestive exposure to a sugar solution US (Kuwabara 1957; Takeda 1961). The US is applied to the bee’s antennae to elicit a proboscis extension, following which it is presented to the proboscis and the bee is allowed to imbibe a small quantity of sugar solution. However, bees also learn to associate the conditioned stimulus (e.g. a neutral odour) with the US when only the antennae are stimulated, although acquisition is slower and memories last only for a few hours (Menzel and Bitterman 1983; Sandoz et al. 2002; Wright et al. 2007). Antennal PER learning assays are useful for separating pre- and post-ingestive processes involved in learning of floral cues and the gustatory assessment of floral rewards (Afik et al. 2006; Wright et al. 2010; Nicholls and Hempel de Ibarra 2013; Simcock et al. 2014, 2018).

We used taste assays and PER conditioning to investigate how honeybees respond during pre-ingestive (antennal) exposure to artificial solutions varying in chemical composition. We predicted that the experimental exposure to sucrose solution artificially contaminated with pollen would result in an increase in proboscis extension and learning performance, relative to sucrose alone. This is due to the fact that adding pollen would increase the concentration of nutritious compounds, such as amino acids, and because bees readily forage on a range of natural nectars containing secondary metabolites (Wright et al. 2013; Tiedeken et al. 2014). However, contrary to our expectations, pollen compounds were found to have a negative effect on bees learning performance. In a series of subsequent investigations, we compared the performance of bees stimulated with pollen–sugar solutions, to those of bees stimulated with sugar solutions containing two compounds known to be aversive to honeybees, sodium chloride (NaCl) and quinine (de Brito Sanchez et al. 2005; Wright et al. 2009, 2010). We also investigated the pre-ingestive responses to sugar solutions supplemented with one of two amino acids, proline and phenylalanine, that have previously been shown to have varying effects, enhancing or inhibiting, on feeding and learning when bees imbibe these solutions (Inouye and Waller 1984; Carter et al. 2006; Simcock et al. 2014). To determine the role of previous foraging experience, we directly compared the responses of pollen and non-pollen foragers, though no differences were observed. Finally, to examine the possibility that non-gustatory cues, such as mechano-reception, play a role in the impairment of learning, we also tested the response of honeybees to a non-nutritive powder, alpha-cellulose, which has previously been used in behavioural assays as a substitute for pollen grains (Waddington et al. 1998; Kitaoka and Nieh 2009; Nicholls and Hempel de Ibarra 2014).

## Methods

Honeybee foragers (*Apis mellifera buckfast)* were collected in individual glass vials from the entrance of queen-right, breeding colonies located at Washington Singer Laboratories, University of Exeter. Departing foragers were collected as is typical for PER conditioning experiments and as in our previous study (Nicholls and Hempel de Ibarra 2013). It should be noted that Experiment 4 only was conducted outside the breeding season. For taste assays, we compared pollen and non-pollen foragers to determine whether prior foraging experience affected their responsiveness. Returning foragers were identified as pollen or non-pollen foragers by the presence or absence of corbicular loads, as in Scheiner et al. (2004). Precautions were taken to avoid collecting young foragers on orientation flights, guard bees that remain at the entrance of the nest, or returning pollen foragers which had lost their corbicular loads and could be mistaken for non-pollen foragers.

To restrain bees for the taste and learning assays, after collection the glass vials were placed on ice and observed, so that immediately after bees stopped moving they could be transferred into metal harnesses which permitted free movement of the antennae and proboscis (Bitterman et al. 1983). Each bee was individually fed 30% (w/w) sucrose solution until satiated and then left undisturbed for 18 or 20 h in a dark, humid box at room temperature until the start of PER conditioning experiments. For the taste assays, bees were not fed but kept for 2 h in the dark.

### Appetitive stimuli

Different compounds were added to a 15% sucrose (S) solution (w/w, 0.44 M) or water to produce stimuli that were applied to the antennae of bees with a toothpick. To prepare a pollen–sucrose stimulus (SPo) for taste assays and PER conditioning experiments, commercial honeybee-collected pollen (Werner Seip, Germany) was ground to a fine powder, and presented in a mixture of varying concentrations (0.1, 1, 10, 30% pollen w/w) with sucrose solution. We also prepared a solution of 30% pollen in water (WPo, w/w, see Nicholls and Hempel de Ibarra (2013)). These solutions were passed through filter paper to remove the larger clumps of pollen which were found to stick to the antennae and interfere with the experiment. We did not observe any clogging of the mouthparts or antennae after repeated stimulation with the filtered solutions containing pollen compounds.

Phenylalanine (SPh), proline (SPr) and quinine hydrochloride (SQ, Sigma Aldrich) were added to 15% sucrose solution at concentrations of 1 mM, 5 mM, 10 mM and 100 mM for taste assays and PER conditioning experiments. To separate the gustatory and mechano-sensory effects of the pollen grains on PER conditioning, the inert, granular substance alpha-cellulose (SCell, 5% w/w) or sodium chloride (SNaCl, 2.5 M) was dissolved in 15% sucrose solution and passed through filter paper.

### Experiment 1: antennal taste assays

We investigated how pollen compounds added to sucrose solutions affected responses towards this stimulus and whether this differed according to forager type (pollen vs. non-pollen foragers). The proboscis extension response of bees to antennal stimulation was recorded for 15% sucrose solution (S) in the first trial and to pollen–sucrose solutions (SPo) of increasing concentrations in trials 2–5 (0.1, 1, 10, 30% pollen w/w). On the final sixth trial, the antennae were stimulated again with 15% sucrose (S), to check that any decline in response to the tested solutions was not the result of a general lack of feeding motivation or fatigue. Between each pollen–sucrose stimulation, the antennae were touched with water. The inter-trial interval (ITI) was 10 min, long enough to avoid sensitisation effects and to distribute conditioning trials sparsely, leading to better acquisition and memory formation (Menzel et al. 1999, 2001). To distinguish between pollen or non-pollen foragers, corbiculae loads of returning foragers were inspected prior to harnessing, and a small piece of tape was added to the base of the restraining harnesses containing pollen foragers. This was hidden from the view of the experimenter, to avoid any potential bias in the coding of behaviour.

Further taste assays were conducted following the protocol above, each with a new group of bees. Sucrose solutions were supplemented with either phenylalanine (SPh), proline (SPr) or quinine (SQ) in increasing concentrations (1 mM, 5 mM, 10 mM and 100 mM) using the same procedures as described above. Finally, we repeated the assay with a further group of bees as a control, applying pure 15% sucrose solution (S) repeatedly over six trials.

### Antennal PER conditioning protocol

In Experiments 2–4, prior to the start of conditioning experiments we tested bees’ antennal sensitivity, to pre-select those individuals that exhibited PER to stimulation with pollen. Bees are known to vary in their responsiveness to pollen (Scheiner et al. 2004) and we wished to rule out a lack of sensitivity to a component of the US as a potential confounding factor in interpreting the results of subsequent learning experiments. The antennae of each subject were first touched with water, with those individuals exhibiting proboscis extension being permitted to drink water until satiated. Antennae were then touched with 30% pollen–water solution, followed by water, 15% sucrose, water again, and finally 30% sucrose, each with a 5-min ITI. The criteria for inclusion in a conditioning experiment were that bees responded to both the 30% pollen–water and 15% sucrose solution (on average, 40% of bees tested at the beginning of each experiment). Following the sensitivity test, bees were randomly assigned to treatment groups. Conditioning began 20 min after the sensitivity test to allow any potential effects of sensitisation to sucrose to subside. When testing bees that were not pre-selected according to their responsiveness to pollen, we still observed that learning was impaired. Therefore to significantly reduce the number of bees used in our experiments, we omitted the pre-selection procedure described above from Experiment 5 onwards.

The conditioned olfactory stimulus (CS) was 1-hexanol (98% purity, Sigma Aldrich) diluted in mineral oil to 2.5 M. Twenty millilitres of the odour solution was placed in a 60-ml glass bottle which was connected to an air pump via silicone tubing (Linander et al. 2012; Wright et al. 2009). The air stream was gated by a valve via a programmable logical controller to deliver uniform odour puffs. The odour stream was directed frontally at the head of the bee and removed in a constant air stream by an extractor system located behind the animal.

An individual bee was placed in the experimental arena at a distance of 4.5 cm from the odour delivery tube and left to acclimatise for 15 s prior to the start of the CS. The odour (CS) was delivered to the antennae for 3 s alone and then overlapping for 1 s with the unconditioned pollen or sucrose stimulus (US). US delivery subsequently continued for a further 2 s. The US was presented to both the right and left antennae. Following US presentation, bees were left in the arena for a further 15 s and then removed for an inter-trial interval of 10 min until the next conditioning trial or test. Proboscis extensions were noted during the presentation of both the CS alone and during US delivery.

### Experiments 2 and 3: establishing the effect of adding pollen compounds to sucrose solutions on the acquisition of a learned olfactory association

In Experiment 2, bees were conditioned over six trials to respond to a neutral olfactory stimulus. The CS was reinforced with one of the following US: 30% (w/w) sucrose solution (30% S), 15% sucrose solution (S), pollen–water (WPo, 30% pollen w/w) or pollen–sucrose solution (SPo, 30% pollen w/w, 15% sucrose). Bees rewarded with 15% sucrose were expected to show a slower rate of acquisition of the conditioned response compared with those receiving 30% sucrose, since sucrose concentration is known to influence learning (Loo and Bitterman 1992). Thus, a comparison against this group permits the detection of any improvement in learning in bees conditioned with a mixture of 15% sucrose and pollen (SPo).

In Experiment 3, we repeated the experiment above (Experiment 2), lowering the concentration of pollen in the SPo solution to 10%, to compare the effects with two different pollen concentrations. The 30% sucrose group was replaced with a group stimulated with water alone (W).

### Experiments 4–6: antennal PER conditioning with sucrose solutions containing a single additional compound

To eliminate the possibility that pollen may impact learning by physically influencing the detection of the CS, for example by clogging the antennal sensilla pores, in Experiment 4 we separated the mechano-sensory effects of the pollen grains from that of their gustatory component using the inert and granular substance, alpha-cellulose as an artificial pollen substitute. This was added to 15% sucrose solution (5% w/w, SCell) and served as the US which was delivered to the antennae. A second group of bees were conditioned with a mixture of salt (NaCl, 2.5 M) and sucrose (SNaCl). While salt is thought to be distasteful to bees when delivered in solution with sucrose at the proboscis, the effect of stimulation at the antennae alone is not known. A third group of bees was stimulated at the antennae with 15% sucrose (w/w) alone (S) and served as a baseline against which to compare performance of bees in other groups.

In Experiment 5, a well-known distasteful compound, quinine, was added to 15% sucrose solution at two different concentrations, 10 mM (10 SQ) and 100 mM (100 SQ). Conditioned responses of bees receiving quinine–sucrose solutions as the US at the antennae were compared with bees receiving either 15% sucrose solution (S) or pollen–sucrose solution (SPo, 30% w/w).

Proline and phenylalanine, two amino acids commonly found in floral nectar and pollen, and previously used in bee choice and learning experiments were chosen to investigate the possible pre-ingestive role of amino acids in the observed effects of pollen in sucrose on PER responses in Experiment 6. Over six training trials, bees in four treatment groups received as the US at the antennae either 15% sucrose alone (S), or 15% sucrose solutions that were supplemented with pollen compounds (SPo, 30% w/w), 100 mM proline (SPr) or 100 mM phenylalanine (SPh).

### Statistical analysis

Statistical analysis was conducted in SPSS 24.0. Subject responses were scored as binary variables, and logit was selected as link function. To scrutinise the learning effects, bees that spontaneously responded to the odour on the first trial of conditioning experiments were excluded from the analysis (Experiments 2–6). Spontaneous response levels were low, no more than 7%, except in Experiment 4 which was conducted during winter, outside the breeding season (18%). GEE (Generalized Estimating Equation) modelling was used to compare the responses in the taste assay across trials with increasing concentrations and the acquisition curves of bees trained with different unconditioned stimuli (Wright et al. 2007). The GEE approach permits a non-normal distribution of the dependent variable and accounts for repeated measurements of the same individual (Hardin and Hilbe 2002). Response to the taste stimulus or to the CS and US in the taste and learning experiments, respectively, was coded as the response variable with treatment and conditioning trial included as factors. Significance tests were based on Wald approximations of the likelihood ratio test. To test whether the highest concentration used in the learning experiments was detectable, we determined for each taste assay the LSD contrast between the first and the fifth trial. Using GEEs, we tested for potential differences between the first and the sixth trial, where bees were exposed to the same condition (pure sucrose solution), and the fifth and the sixth trials (between the highest concentration and the final sucrose test). In the learning experiments, GEEs in combination with contrast analysis using LSD tests compared each treatment group to the control. Differences between treatment groups in the last conditioning trial and unrewarded tests were compared using GZLMs.

## Results

### Experiment 1: pre-ingestive responses to sucrose solutions containing nutritional and aversive compounds in pollen and non-pollen foragers

**Fig. 1:**
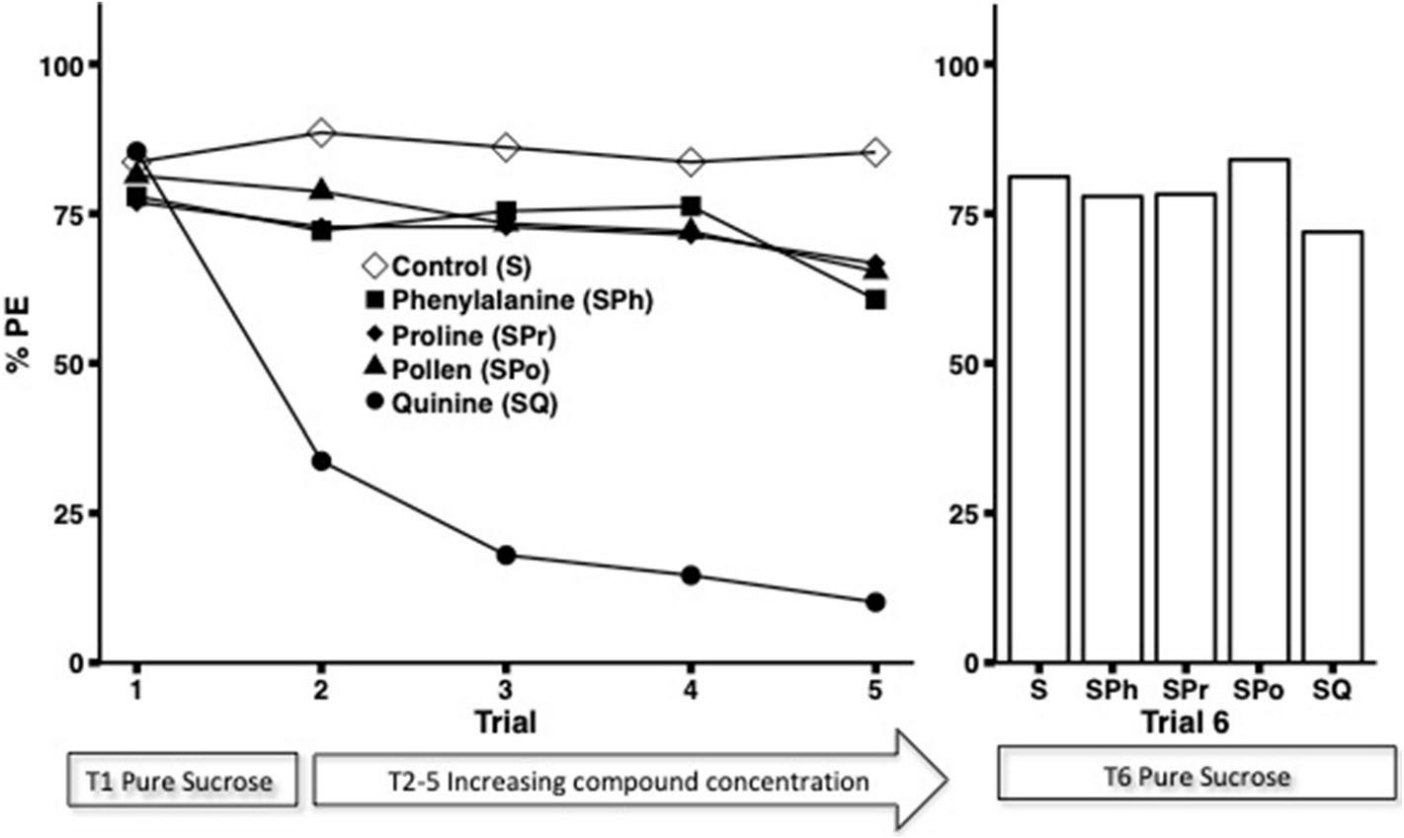
Antennal taste assays (Experiment 1). The percentage of bees responding with PER to antennal stimulation over 6 trials, starting and ending with the application of 15% sucrose solution (trials 1 and 6). Control bees (*N* = 132) received the 15% sucrose solution in all trials, whilst in the other groups bees were tested in trials 2–5 with solutions that were supplemented with, phenylalanine (SPh, *N* = 130), pollen compounds (SPo, *N* = 90), proline (SPr, *N* = 156), and quinine (SQ, *N* = 122). The concentration of the compounds was increased in ascending order (1 mM, 5 mM, 10 mM, 100 mM; or for SPo 0.1%, 1%, 10%, 30%)

When bees’ antennae were stimulated with sucrose solutions supplemented with pollen (SPo) or the amino acid phenylalanine (SPh), the proportion of proboscis extensions (%PE) declined significantly with increasing concentration (Fig. 1, Table 1(1), (3)). In both groups, the percentage of bees responding differed significantly between the first (pure sucrose) and fifth trial (100 mM Spo or SPh) (LSD, SPo, *χ*^2^ = 5.61, *p* = 0.018, SPh, *χ*^2^ = 10.57, *p* = 0.001). However, responsiveness to pure sucrose did not change over the course of the experiment, (T1–T6, SPo, *df* = 1, *χ*^2^ = 0.17, *p* = 0.68; SPh, *df* = 1, *χ*^2^ < 0.0001, *p* = 1.0).

**Table 1:** GEE analysis of responses in the taste assays (Experiment 1)

	Experiment	Factor	*df*	*X* ^2^	*P*
1	SPo	**Trial**	**4**	**10.11**	**0.039**
		Forager type	1	0.19	0.66
		Trial × forager type	4	8.02	0.091
2	SPr	Trial	4	5.76	0.22
		Forager type	1	0.68	0.41
		Trial × forager type	4	2.07	0.72
3	SPh	**Trial**	**4**	**12.77**	**0.012**
		Forager type	1	0.17	0.68
		Trial × forager type	4	0.21	0.99
4	SQ	**Trial**	**4**	**77.41**	**< 0.001**
		Forager type	1	0.13	0.72
		Trial × forager type	4	8.22	0.084
5	S	Trial	4	3.72	0.45
		Forager type	1	0.42	0.52
		Trial × forager type	4	3.03	0.55

Factors in bold indicate a significant effect on PER (*p* < 0.05)

As expected, the strongest decrease in responses with increasing stimulus concentration was found for sucrose solutions containing the known aversive compound quinine (SQ, Fig. 1, Table 1(4)). The proportion of bees responding fell significantly between the first (pure sucrose) and fifth trial (100 mM, LSD, *χ*^2^ = 247.27, *p* < 0.001). Though for this group there was a significant difference between the response to pure sucrose at the beginning and end of the taste assay (T1–T6, *df* = 1, *χ*^2^ = 5.1, *p* = 0.024), close to 75% of bees exhibited proboscis extensions on the final trial. This suggests that bees were still very motivated to respond to pure sucrose, compared to the sucrose–quinine mixture (T5–T6, *df* = 1, *χ*^2^ = 39.55, *p* < 0.001).

In contrast to the results obtained for pollen and phenylalanine, bees stimulated at the antennae with sucrose supplemented with the amino acid proline (SPr) did not show a significant change with increasing proline concentration (Table 1(3)), although there was a strong tendency to not respond at the highest concentration of 100 mM (Fig. 1, Trial 5), and the percentage of bees responding on this fifth trial was significantly lower than on the first trial when bees were stimulated with pure sucrose solution (LSD, *χ*^2^ = 5.67, *p* = 0.017). When responses to pure sucrose solution were re-tested on the final trial, there was a significant increase in response compared to the previous trial with the highest concentration of proline, suggesting bees were able to distinguish between the two solutions and that the presence of high concentrations of proline negatively impacted their motivation to respond (T5–T6, *df* = 1, *χ*^2^ = 9.6, *p* = 0.002). Comparing responses to pure sucrose solution between the first and final trial, no statistically significant difference was found (T1–T6, *df* = 1, *χ*^2^ = 0.38, *p* = 0.54).

As expected, bees in the control group showed no decline in proboscis extensions over the course of five trials of repeated antennal stimulation with 15% pure sucrose (S) solution (Fig. 1, Table 1(5)), further suggesting that any decline in response to increasing concentrations of compounds exhibited by the other groups of bees was not simply the result of fatigue. On average, 85% of bees responded to sucrose stimulation at the antennae, and there was no significant difference in response level between the first and fifth (LSD, *χ*^2^ = 0.29, *df* = 1, *p* = 0.59), first and sixth (*χ*^2^ = 0.11, *df* = 1, *p* = 0.74) or fifth and sixth trials (*χ*^2^ = 0.88, *df* = 1, *p* = 0.35).

In all groups, except SQ, the response to water increased over time (Table S1). In the SQ group, the proportion of proboscis extensions remained low throughout the experiment fluctuating between 0 and 12%, in SPo it increased moderately between 7% and 19%. In the other three groups, responses went up from 5–8% to 36–38%. Bees were not pre-fed water like in some other studies (e.g. Pankiw et al. 2001) and some might have been thirsty as response levels came close to those found in water collecting foragers that were stimulated with pure water (around 30–60% PE, Lau and Nieh 2016). But importantly, we did not see an increase in response to sucrose in the control (S) group over time. Furthermore, the inter-trial interval of 10 min was long, and work in *Drosophila* suggests that in insects neural circuits regulating thirst and responses to water are likely to be separate from those activated by sucrose (Lin et al. 2014). We, therefore, assume that the bees’ responses to test solutions were not affected by the changes in water responsiveness.

No differences in the gustatory responses were found between pollen and non-pollen foragers. It has been shown previously that pollen foragers can be more sensitive to sucrose than non-pollen foragers (Page et al. 1998; Pankiw and Page 2000; Scheiner et al. 2001, 2004), however, response levels can vary with changes in environmental factors such as floral resource availability, crop filling and foraging experience (Pankiw et al. 2001; Scheiner et al. 2003). The question whether pollen foragers are more or less sensitive to pollen compounds in sugar solutions should be explored further.

### Experiment 2: does adding pollen compounds to sucrose solutions affect the acquisition of a learned olfactory response?

**Fig. 2:**
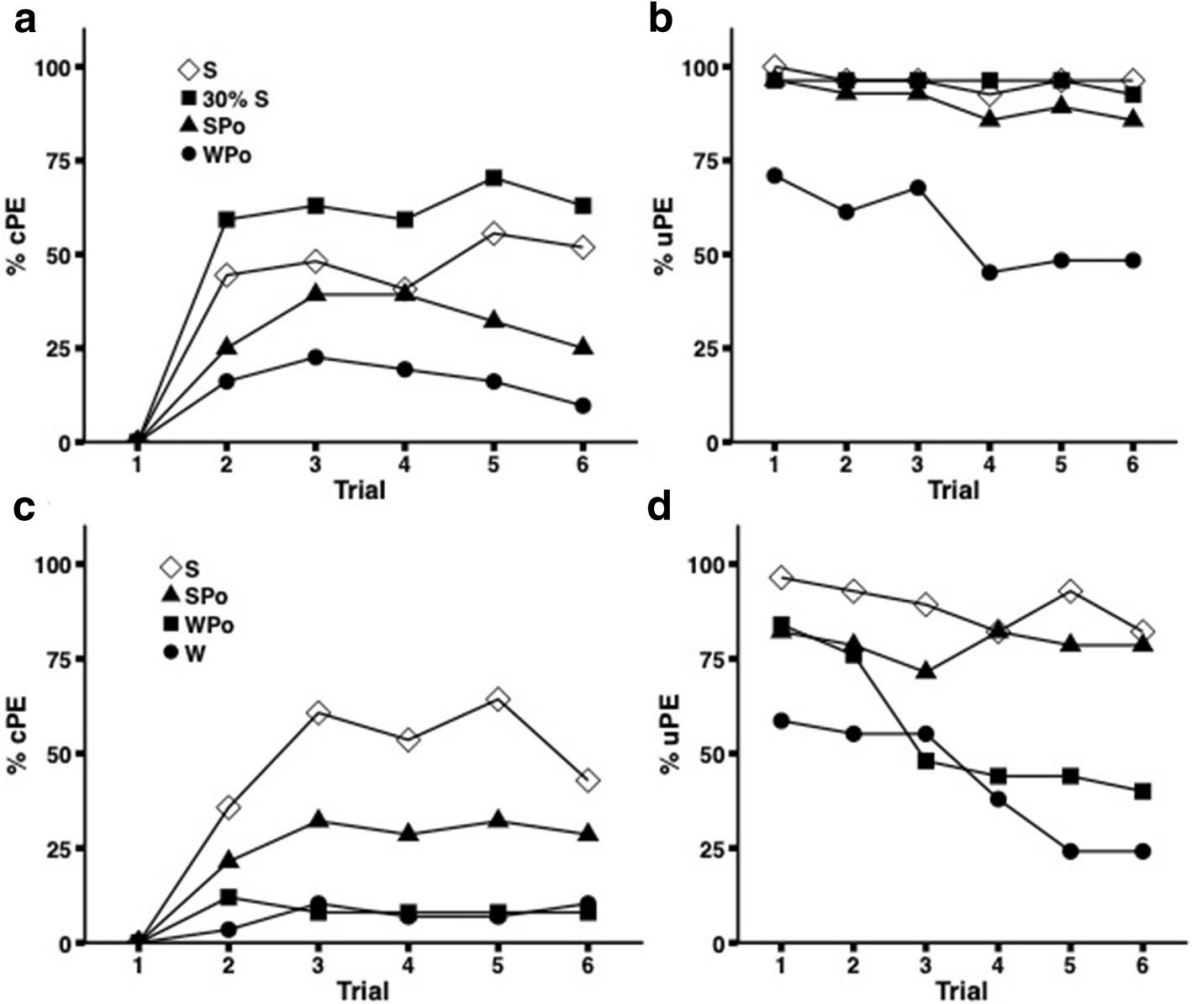
Conditioning with pollen-supplemented sucrose solution. Shown are responses to the presentation of the CS (left) and US at the antennae (right). **a** In Experiment 2, bees were conditioned with either sucrose (S, *N* = 27, or 30% S,* N* = 27), pollen-supplemented sucrose solution (SPo, *N* = 28), or as a negative control with pollen-supplemented water solution (WPo,* N* = 31) which does not reinforce learning (Nicholls and Hempel de Ibarra 2013). **b** In Experiment 3, less concentrated SPo (*N* = 28) was used and compared to water (W,* N* = 29), pollen-supplemented water (WPo,* N* = 25) and sucrose (S,* N* = 28)

We used the olfactory PER conditioning paradigm to test whether supplementation of the typical sucrose unconditioned stimulus (US) with pollen leads to a change in learning performance. Initially, we conducted experiments in which the US was applied to both the antennae and proboscis but observed that the addition of pollen led to lower response rates (Fig S3) which could be due to post-ingestive processes. Therefore, the US was delivered to the antennae only in all subsequent experiments. The US applied was pollen–sucrose solution (SPo), pollen–water solution (WPo) or pure sucrose solution (either S or 30% S). The type of US had a significant effect on the overall level of acquisition (Fig. 2a, Table 2(1)). The lowest level of conditioned proboscis extension responses (cPE) was recorded for bees experiencing the WPo solution, replicating our previous finding that this US does not lead to a conditioned response to olfactory stimuli (Nicholls and Hempel de Ibarra 2013). When the US contained pollen and sucrose bees showed somewhat higher responses, but still lower than bees stimulated with sucrose alone (S). As expected, bees rewarded with 30% S showed the highest level of acquisition. Although the GEE showed a significant effect for treatment, contrast analysis revealed no difference in acquisition between the S and SPo groups (LSD, *p* = 0.113) showing that the addition of pollen did not lead to an improvement in learning. In fact, from the fifth trial onwards, bees reinforced with the SPo solution showed a decline in response to the CS, relative to those receiving the 15% sucrose alone (S). Analysis of the final training trial reveals that the response of bees in the SPo group was significantly lower (GZLM, Trial 6 *χ*^2^ = 18.63, *df* = 3, *p* < 0.001, LSD, S vs. SPo, *p *= 0.033).

**Table 2:** GEE analysis of the CS responses (cPE) in the antennal conditioning experiments (Experiments 2–6)

	Experiment	Factor	*df*	*X* ^*2*^	*P*
1	Experiment 2 S, S (30%), SPo, WPo	Trial	4	5.93	0.20
		**Treatment**	**3**	**25.07**	**<0.001**
		Trial × Treatment	12	11.85	0.46
2	Experiment 3 S, SPo (10%), WPo, W	Trial	4	5.099	0.28
		**Treatment**	**3**	**34.62**	**<0.001**
		Trial × Treatment	12	12.61	0.398
3	Experiment 4 S, SCell, SNaCl	**Trial**	**4**	**36.37**	**<0.001**
		Treatment	2	4.69	0.096
		Trial × Treatment	8	3.04	0.93
4	Experiment 5 S, SPo, SQ (10 mM), SQ	**Trial**	**4**	**19.11**	**0.001**
		**Treatment**	**3**	**43.69**	**<0.001**
		**Trial** × **Treatment**	**12**	**40.05**	**<0.001**
5	Experiment 6 S, SPo, SPh, SPr	**Trial**	**4**	**59.77**	**<0.001**
		Treatment	3	3.78	0.29
		Trial × Treatment	12	20.22	0.063

Factors in bold indicate a significant effect on cPE (*p* < 0.05)

It may be that the apparent decline in proboscis extension to the CS (cPE) when conditioned with SPo solutions resulted from lower rates of proboscis extension to the US (uPE). Therefore, we analysed differences between groups in terms of PE during the US application at the antennae (Fig. 2b, Table 3(1)). On the final conditioning trial, a significant difference in level of responding to the US was observed (GZLM, Trial 6 *χ*^2^= 20.124, *df* = 3, *p* < 0.001), driven by the low level of responding in bees stimulated with the WPo solution. No difference between the SPo and S groups was observed (LSD, *p* = 0.161), though again a similar trend of a slight, but steady, decrease in cPE response to SPo was observed. Similar results were obtained when bees were moderately satiated prior to the onset of the experiment (Fig. S4).

**Table 3:** GEE analysis of the US responses (uPE) in the antennal conditioning experiments (Experiments 2–6)

1	Experiment 2 S, S (30%), SPo, WPo	Trial	4	8.18	0.085
		**Treatment**	**3**	**31.28**	**<0.001**
		Trial × Treatment	12	15.61	0.21
2	Experiment 3 S, SPo (10%), WPo, W	**Trial**	**5**	**19.71**	**0.001**
		**Treatment**	**3**	**27.68**	**<0.001**
		**Trial** × **Treatment**	**15**	**34.32**	**<0.003**
3	Experiment 4 S, SCell, SNaCl	N/A			
4	Experiment 5 S, SPo, SQ (10 mM), SQ	**Trial**	**5**	**53.03**	**<0.001**
		**Treatment**	**3**	**28.74**	**<0.001**
		**Trial** × **Treatment**	**15**	**25.58**	**0.043**
5	Experiment 6 S, SPo, SPh, SPr	**Trial**	**5**	**36.77**	**<0.001**
		Treatment	3	3.12	0.37
		Trial × Treatment	15	11.83	0.69

Factors in bold indicate a significant effect on uPE (*p* < 0.05)

HPLC analysis revealed that WPo solutions had significant concentrations of sugars, on average 0.4 M glucose and 0.5 M fructose (Fig. S1, for methods see supplemental materials). The concentrations of 21 proteinogenic amino acids were also quantified, of which 10 are known as essential amino acids (EAAs) for bees (de Groot 1953 as cited in Paoli et al. (2014), Fig S1). The sugar content was higher in SPo solutions, on average 1.1 M glucose and 1.2 M fructose. However, the hydrolysis of sucrose cannot explain our findings for WPo and SPo solutions. For instance, fructose and glucose are strong phagostimulants, though weaker than sucrose (Afik et al. 2006; Simcock et al. 2018). Furthermore, we found that bees learned significantly better in antennal PER conditioning (Fig. S2) where the US contained 1 M glucose and 1 M fructose than with 15% sucrose (S) solution (0.44 M).

### Experiment 3: is the inhibition of learning dependent on the concentration of pollen added to sucrose?

When the concentration of pollen in the unconditioned stimulus was reduced from 30% to 10% (w/w) in 15% sucrose, bees still performed more poorly than those rewarded with 15% sucrose alone, thus even at weaker concentrations pollen compounds inhibited cPE to olfactory stimuli (Fig. 2c, Table 2(2), LSD S vs. SPo, * p* = 0.01, GZLM Trial 6  *χ*^2^ = 10.95, *df* = 3, *p* = 0.012). Bees in the 10% SPo group showed a higher level of acquisition than those conditioned with WPo and Water as US, with the latter exhibiting cPEs at a level corresponding to the spontaneous probability (8.8%). The uPE responses differed more markedly across groups, even though the level of uPEs to SPo remained high and not statistically different from the control group (Fig. 2d, Table 3(2), LSD S vs. SPo, *p* = 0.18).

### Experiment 4: separating the chemical and mechano-sensory effects of pollen compounds in sucrose solutions

**Fig. 3:**
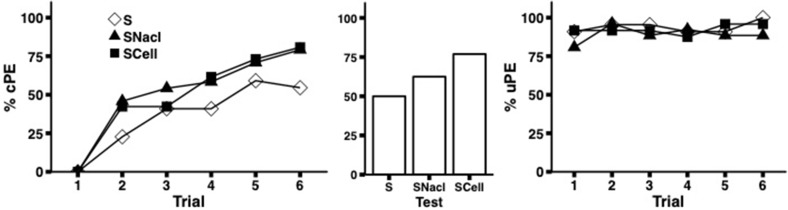
Separating a potential mechanical effect. Bees were conditioned with either sucrose solution (S, *N* = 22), a mix of sucrose and salt (SNaCl, *N* = 24) or sucrose solution supplemented with alpha-cellulose (SCell, *N* = 26). The left panel shows the acquisition curves for the CS responses and the right panel the PER extensions when presenting the US to the antennae during the conditioning trials. The middle panel depicts the performance in the final unrewarded test

Supplementing 15% sucrose solution with either salt (NaCl) or alpha-cellulose had no negative impact on the rate or overall level of acquisition (Fig. 3). On the contrary, bees experiencing reinforcement with these mixtures tended to outperform those in the control group, however group differences were not statistically significant (Table 2(4), GZLM Trial 6 *χ*^2^ = 4.72, *df* = 2* p* = 0.095, GZLM Test  *χ*^2^ = 3.64, *df* = 2, *p* = 0.16). All bees showed consistently very high uPE response to antennal stimulation with their respective US over the course of training (Fig. 3, Table 3(3), due to a lack of maximum likelihood estimates GEE and GZLMs were not conducted). The marginally better performance of bees receiving a mixture of salt and sucrose can most likely be explained by the higher molarity of sugar in this solution relative to pure sucrose solution (0.64 M compared to 0.5 M). Since alpha-cellulose is not soluble, the elevated performance of bees in this group is more difficult to account for.

### Experiment 5: comparing the effect of pollen compounds to that of a known aversive stimulus, quinine

**Fig. 4:**
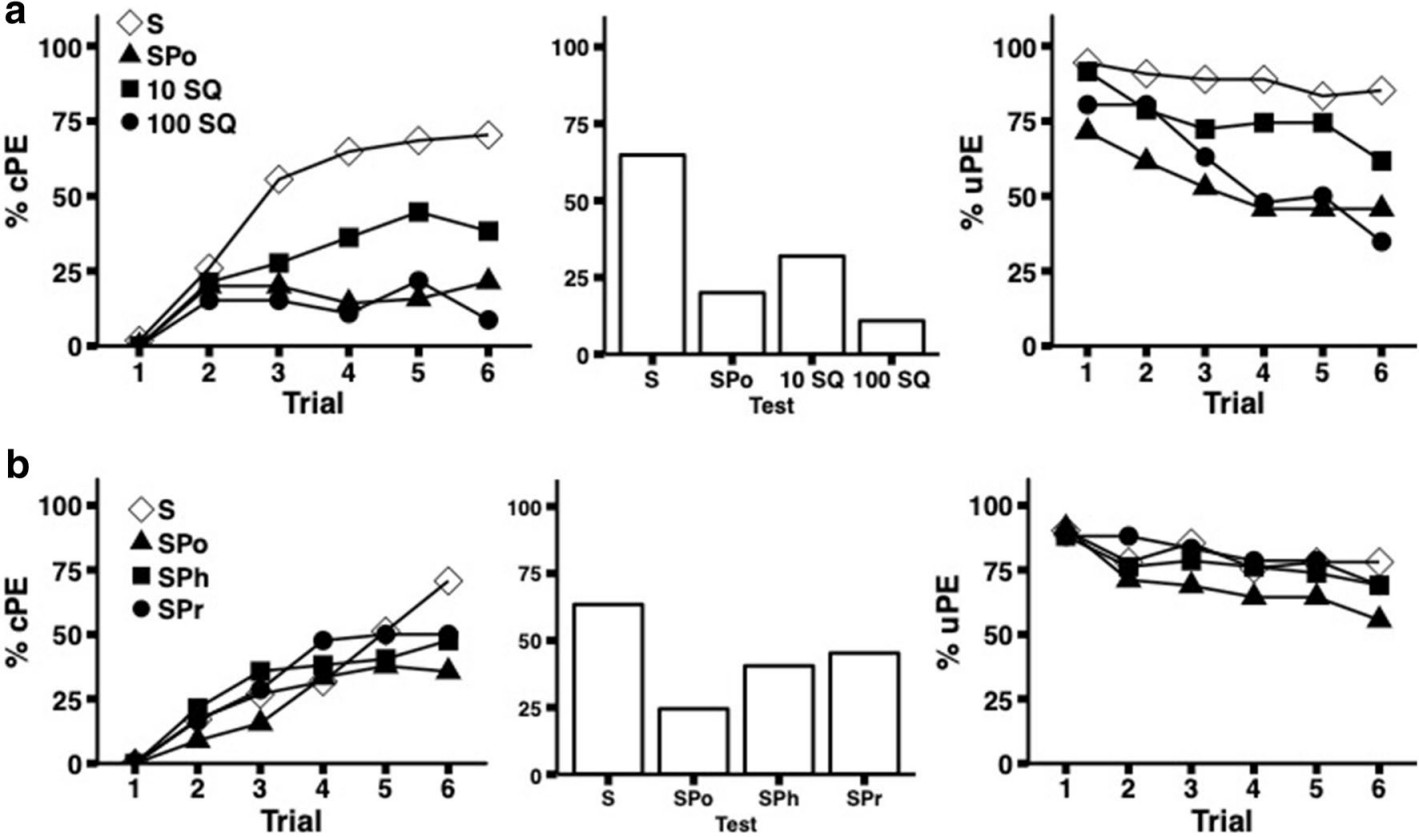
Antennal PER conditioning with solutions containing proline, phenylalanine, quinine or pollen compounds. The left panels shows the acquisition curves for the CS responses and the right panels the PER extensions when presenting the US to the antennae during the conditioning trials. The middle panel shows the CS responses in the final unrewarded test. **a** Experiment 5: shown are the experimental results for bees conditioned with either 15% sucrose solution (S, *n* =  54), or solutions supplemented with pollen compounds (SPo, *n* = 70), or quinine (10 mM SQ, *n* = 47; 100 mM SQ, *n* = 46). **b** Experiment 6: bees were presented with sucrose solution (S, *n* = 41), or solutions supplemented with pollen compounds (SPo, *n* = 45), 100 mM phenylalanine in 15% sucrose solution (SPh, *n* = 42) or 100 mM proline (*n* = 42) as US

We tested whether the inhibitory effect of pollen compounds in sucrose solutions was comparable to that of a known aversive compound, quinine. There was a significant difference in the levels of acquisition between the groups (Fig. 4a, Table 2(4), GZLM Trial 6 *χ*^2^ = 41.02, *df* = 3, *p* < 0.001). Contrast analysis revealed a significant difference between cPE responses of bees rewarded at the antennae with pure sucrose solution (S), compared to the three other groups (LSD SQ10 vs. S, *p* = 0.003, SPo vs. S and SQ100 vs. S, *p* < 0.001). The SQ10 bees showed a better rate of acquisition than the SQ100 group, and the SPo group exhibiting an intermediate rate. The differences in acquisition were mirrored in the uPE responses when applying sucrose solution containing either quinine or pollen as a US (Fig. 4a, Table 3(4), LSD S vs. SPo and S vs. SQ100, *p* < 0.001, GZLM Trial 6  *χ*^2^ = 26.73, *df* = 3, *p* < 0.001). The unrewarded test yielded the same results, with a significant difference in cPE between bees conditioned with sucrose alone and the three other groups, but no difference in the SPo or SQ groups (Fig. 4a, GZLM Test *χ*^2^ = 35.19, *df* = 3, *p* < 0.001, LSD  S vs. SPo, S vs. SQ10, S vs. SQ100, *p* < 0.001).

In comparison to Experiments 2 and 3, bees stimulated with a mixture of pollen in sucrose (SPo) showed a stronger decline in uPE response to the US and weaker acquisiton of cPE responses over the course of the experiment as compared to the control group. For instance, just 46% of bees exhibited uPE responses on the sixth trial, compared to 71% on the first trial, with cPE aquisition reaching a level of just above 20% (well above the spontaneous response probability of 4.8%), which suggestively points towards a link between effectiveness of the US and strength of learning.

### Experiment 6: antennal PER conditioning with sucrose solutions containing amino acids

Supplementation of the sucrose solution with either pollen or amino acids did not affect the rate of acquisition relative to those bees receiving pure sucrose solution (Fig. 4b, Table 2(5)). The performance was less varied than in Experiment 5, importantly, however, bees in the sucrose–pollen group (SPo) had again the lowest level of cPE responses. As in previous experiments, by the final trial, the cPE response of bees stimulated with SPo was significantly lower than in the control (S) (GZLM Trial 6 *χ*^2^ = 10.34, *df* = 3, *p* = 0.016, LSD S vs. SPo, *p* < 0.001, S vs. SPh, *p* =  0.027, S vs. SPr, *p* = 0.048). Furthermore, during the unrewarded test, there was a significant effect of US type, with bees in the SPo group showing the lowest level of responses after acquisition, and bees in the control (S) the highest (GZLM Test  *χ*^*2*^= 12.72, *df* = 3, *p* = 0.005, LSD S vs. SPo, *p* < 0.001, S vs. SPh, * p* = 0.032). Bees receiving a mixture of sucrose and either the amino acid phenylalanine (SPh) or proline (SPr) exhibited an intermediate level of acquisition and test responses compared to the  S and SPo groups. In terms of responsiveness to the delivery of the US, all treatment groups displayed similarly high uPE response to antennal stimulation that declined to some but equal extent in all groups over the course of the experiment (Fig. 4b, Table 3(5)).

## Discussion

Flowers offer pollinators appetitive rewards with complex chemical compositions, yet the functional consequences of this diversity in reward composition for the behaviour of pollinators are still to be fully understood (for review see Stevenson et al. 2017; Nicholls and Hempel de Ibarra 2017; Wright et al. 2018). The role of antennal sensory signals in tasting compounds, reinforcing behaviour and eliciting feeding responses is also little explored, though bees actively deploy their antennae when foraging on flowers. Sensory signals from the antennae form part of the neural circuit which controls the extension of the proboscis for feeding (Menzel et al. 1999), and thus directly influence decision-making processes in foraging bees.

We expected that enriching sugar–water solutions with additional nutrients, either via mixtures of pollen compounds or the addition of single amino acids (proline or phenylalanine), would increase gustatory and learned responses in bees. However, in contrast we found that the addition of pollen to the sucrose solution led to less frequent proboscis extension responses during taste assays and US presentation, and a reduced acquisition of a conditioned olfactory response compared to bees that received sucrose alone. We ruled out the effect of mechano-sensory cues and the ‘clogging’ of antennal sensilla pores by showing that acquisition was unaffected by the addition of an inert granular substance, alpha-cellulose. Therefore, the most parsimonious explanation is that the sucrose solution with pollen compounds was more distasteful, which reduced its reinforcing potential (e.g. Loo and Bitterman 1992, Wright et al. 2010) and consequently detracted from its subjective reward value, as compared to pure sucrose.

The effect was observed regardless of whether bees were stimulated at the antennae only or also permitted to imbibe the US, suggesting that pollen compounds can be detected pre-ingestively at the antennae. When the concentration of pollen added to the sucrose solution was reduced from 30% to 10%, such inhibition of learning was still observed. Indeed, the learning performance of bees stimulated with pollen–sucrose solutions as US was similar to those presented with solutions containing bitter-tasting quinine, a substance known to be aversive to bees (de Brito Sanchez et al. 2005; Wright et al. 2010; Kessler et al. 2015). Thus, it seems likely that pollen may also contain compounds that are perceived as distasteful by bees, which consequently impair learning performance.

Previous work has shown that the application of pollen to the antennae, either in dry form or mixed with water, elicits unconditioned proboscis extension (Scheiner et al. 2004; Nicholls and Hempel de Ibarra 2013) Thus, pollen can elicit a feeding response. Also, during pollen collection bees have been shown to learn floral cues (Nicholls and Hempel de Ibarra 2014; Russell et al. 2016; Muth et al. 2016). However, pollen does not lead to conditioned proboscis extension (cPE) in a classical conditioning paradigm (Nicholls and Hempel de Ibarra 2013). It appears that no single nutrient type, other than sugar, is sufficient alone in reinforcing the conditioned response to a neutral odour. Similarly, when discriminating between the nutritional quality of pollen samples using taste, the presence of sugar for reinforcement seems to be required (Ruedenauer et al. 2015, 2018). Our present findings further suggest that the strength of learning is contingent on how strongly sucrose masks the presence of distasteful compounds.

Chemical analysis revealed that the pollen-supplemented samples were highly rich in amino acids, which may explain why these samples were perceived as distasteful by bees. While some amino acids have been shown to enhance feeding rates and olfactory conditioning (Alm et al. 1990; Kim and Smith 2000; Carter et al. 2006; Petanidou et al. 2006; Wright et al. 2009), this effect is known to be concentration dependent and some amino acids, such as serine, have been shown to adversely affect the feeding rate of bees (Inouye and Waller 1984; Bertazzini et al. 2010). For instance, when feeding honeybees under conditions similar to those in our study, Simcock et al. (2014) showed that bees would feed less on sucrose solutions containing 100 mM of proline, phenylalanine and other single amino acids. However, in those experiments the amino acid solutions were only presented to the proboscis, while the antennae were stimulated with pure sucrose solution. Where a positive effect of proline on olfactory conditioning has been observed (Wright et al. 2009, Simcock et al. 2014), because bees were permitted to imbibe the US, this effect may have been mediated by sensory signals from the proboscis, or could have arisen from post-ingestive processes.

While pollen constitutes the main source of amino acids for bees, it also contains various other nutrients, such as proteins, lipids and fatty acids (Singh et al. 1999; Schmidt and Hanna 2006; Avni et al. 2014; Vaudo et al. 2016), as well as secondary compounds including non-protein amino acids, alkaloids, phenols and glycosides (discussed by Nepi 2014; Nicolson 2011; Wright et al. 2018). The occurrence of secondary metabolites in floral pollen and nectar is thought to play a role in deterring herbivores and has demonstrated ecological benefits for flowering plants (Adler and Irwin 2005; Stevenson et al. 2017). Though such compounds are toxic at high concentrations, they are nevertheless accepted by foragers of some bee species when present in naturally occurring, low concentrations (Wright et al. 2013; Tiedeken et al. 2014, 2016). The pollen used in the current study was honeybee collected, thus the lack of palatability may at first appear paradoxical. Although honeybee foragers are unlikely to directly assess the nutritional quality of pollen during collection, given they do not typically ingest this reward whilst foraging, their gustatory organs, the antennae, proboscis and tarsi, all frequently come into contact with pollen during collection (reviewed by Nicholls and Hempel de Ibarra 2017). This means bees have ample opportunity to obtain taste-related information and possibly avoid collecting toxic pollen, perhaps similar to toxic nectars (Barlow et al. 2017). However, it is possible that in the process of preparing our stimuli, where dry pollen was mixed with sucrose solution, the pollen grains were placed under osmotic shock potentially releasing some compounds into solution which a pollen-foraging honeybee would not typically be exposed to. Thus, we cannot conclude with certainty which pollen compounds contributed to the adverse effect on learning and gustatory responses observed in our study.

The gustatory repertoire of honeybees is often considered to be limited, given that relative to other insects such as flies, few gustatory receptor genes have been identified. While it has been suggested that this reflects the narrow breadth of the honeybee diet (Robertson and Wanner, 2006), the current findings, coupled with further recent advancements in understanding of nectar chemistry, suggest that nectars of different plants and flowers may vary more strongly in their taste for bees than hitherto thought. The specific contribution of the antennae to the gustatory assessment of nectars is still little understood. At least ten types of gustatory receptors are expressed in the honeybee antennae (Simcock et al. 2017) including sugar receptors GR1 and GR2 (Jung et al. 2015). While a dedicated gustatory receptor for bitter tastes has yet to be identified (de Brito Sanchez et al. 2005), there are some putative candidates (Robertson and Wanner 2006). Wright et al. (2010) have shown that quinine elicits a specific pattern of neuron firing across gustatory receptors, suggesting that each receptor may be widely tuned to a variety of gustatory compounds, with unique patterns of activation/inhibition coding for individual compounds.

An electrophysiological study of the chemoreceptors found on the mouthparts of hoverflies (*Eristalis tenax*), whose ecology closely resembles that of bees, found that extracts of pollen in water stimulate the salt receptor cell (Wacht et al. 2000). The learning performance of bees reinforced with SPo was, therefore, compared with that of bees conditioned with a salty sucrose solution (SNaCl). We found that bees in the SNaCl group performed as well as those stimulated with sucrose alone, which was surprising given that nectars containing salts are known to be less attractive to free-flying bees (von Frisch 1942; Waller 1972) and studies have used salt–water solutions applied to the antennae as an aversive reward in learning experiments (Wright et al. 2009; Linander et al. 2012). In fact in our study, bees stimulated with salt–sucrose mixtures showed a tendency to slightly outperform sucrose-only bees, likely owing to the slightly higher sucrose molarity as compared to the pure solution or potentially because in hungry bees the appetitive effect of the sugar outweighs the aversive effect of the salt.

It is only relatively recently that the effect of compounds other than sugars on pollinator learning and foraging decisions have been investigated. Aside from deterring herbivory and nectar robbing, distasteful compounds have been postulated to benefit plants by limiting the drinking time of individual foragers, facilitating movements between flowers and improving pollen transfer (Kessler et al. 2008). However, it is important also to consider the possibility that pollinators may learn to recognise such flowers on the basis of distasteful cues and avoid these flowers altogether. For example, Adler and Irwin (2005) observed that increasing the concentration of the alkaloid gelsemine in *Gelsemium sempervirens* reduced nectar consumption, but also floral visits and pollen transfer. It could protect flowers from damage caused by over visitation or exclude certain species of pollinators. Growing evidence indicates that plants have evolved strategies to adaptively shift the fine balance between attraction and deterrence for controlling pollinator behaviour. Our study supports the view that variations in composition of floral rewards have important effects on pollinators. Further studies of how the antennae process gustatory information and how pre-ingestive signals mediate learning and decision making will help to understand how bees assess and respond to varying qualities of floral rewards.

## Electronic supplementary material

Below is the link to the electronic supplementary material.

Supplementary material 1 (PDF 691 kb)

## Abbreviations

cPE: Conditioned proboscis extension
CS: Conditioned olfactory stimulus
GEE: Generalized estimating equation
ITI: Inter-trial interval
LSD: Least significant difference
PE: Proboscis extension
PER: Proboscis extension response
S: Sucrose solution
SCell: Sucrose solution with cellulose
SPh: Sucrose solution with phenylalanine
SPo: Sucrose solution with pollen compounds
SPr: Sucrose solution with proline
SQ: Sucrose solution with hydrochloride
uPE: Unconditioned proboscis extension
US: Unconditioned stimulus
W: Water
WPo: Pollen compounds in aqueous solution
