# Supplementary material for: A matter of taste: the adverse effect of pollen compounds on the pre-ingestive gustatory experience of sugar solutions for honeybees
Source: J Comp Physiol A Neuroethol Sens Neural Behav Physiol. 2019 Jun 5;205(3):333–46. doi: 10.1007/s00359-019-01347-z (PMC6579781; doi:10.1007/s00359-019-01347-z)
Supplement: Supplementary file 1 — Supplementary material 1 (PDF 691 kb) [file 359_2019_1347_MOESM1_ESM.pdf]

Supplemental Materials

A matter of taste: the adverse effect of pollen compounds on the pre-ingestive gustatory experience of sugar solutions for honeybees

E. Nicholls[1,2], S. Krishna[1,3*], O. Wright[1*], D. Stabler[4], A. Krefft[1], H. Somanathan[3], N. Hempel de Ibarra[1&]

[1] Centre for Research in Animal Behaviour, University of Exeter, UK
[2] Present address: School of Life Sciences, University of Sussex, UK
[3] Centre for Research in Ecology and Evolution, Indian Institute of Science Education and Research Thiruvananthapuram (IISER-TVM), India
[4] Institute of Neuroscience, University of Newcastle, UK
[*] contributed equally
[&] Correspondence to: N.Hempel@exeter.ac.uk

HLPC analysis

Carbohydrate analysis

The concentrations of glucose, fructose, sorbitol and sucrose in treatment solutions were quantified using high performance ion chromatography (HPIC). Following pilot analyses, samples were diluted 1 in 2000 so that the concentration of sugars were within a reliable range of detection for the machine. For each sample, 30 µl was pipetted in to an analysis vial. From this sample, 20 µl was injected on to a Carbopac PA-100 column (Dionex, Sunnyvale, California, USA) fitted with a Dionex Carbopac PA-100 BioLC guard (4 x 50 mm). Elution was run isocratically using helium de-gassed 100 mM NaOH as the mobile phase. Flow rate was set at 1 ml min^{-1} for 10 min at room temperature. Pulsed amperometric detection recorded the chromatographic profile using an ED40 electrochemical detector (Dionex, Sunnyvale, California, USA). Chromatographic profiles were analysed using Chromeleon v.6.8 software (Thermo Fisher Scientific Inc., MA, USA). Concentrations of sugars in the test solutions were calculated by comparison to a reference standard of each sugar/sugar alcohol.

Amino acid analysis

Amino acid concentrations in test solutions were quantified using ultra high-performance liquid chromatography (UHPLC) Ultimate 3000 system. Pilot analyses revealed that the optimal dilution for detection of amino acids was 1 in 30 of the filtered test solutions. Solutions were passed through 0.45 µm syringe filter tips before 10 µl of sample was added to an analysis vial. Amino acids were derivatised pre-column using a pre-programmed autosampler (Ultimate 3000 Autosampler, Dionex, Thermo Fisher Scientific Inc.). The 10 µl of sample was treated for 1 min with 15 µl of 7.5 mM o- phthaldialdehyde (OPA) and 225 mM 3- mercaptopropionic acid (MPA) in 0.1 M sodium tetraborate decahydratre ($Na_2B_4O_7 \cdot 10\ H_2O$), pH 10.2 and for 1 min with 10 µl of 96.6 mM 9-fluroenylmethoxycarbonyl chloride (FMOC) in 1 M acetonitrile. This was followed by the addition of 6 µl of 1M acetic acid. After pre-treating, 30 µl of the amino acid derivatives were then injected onto a 150 x 2.1 mm Accucore RP-MS (Thermo Fisher Scientific Inc.) uHPLC-column. Elution

solvents were: A = 10 mM di-sodium hydrogen orthophosphate (Na_2HPO_4), 10 mM

$Na_2B_4O_7 \cdot 10\ H_2O$, 0.5 mM sodium azide ($NaN_3$), adjusted to pH 7.8 with concentrated HCl, and B = Acetonitrile/Methanol/Water (45/45/10 v/v/v). Flow rate was set at 500 µl min using a linear gradient of 3 to 57% (v/v) of solvent B over 14 min, followed by 100% solvent B for 2 min and a reduction to 97% solvent B for the remaining 4 min. Derivatives were detected by fluorescence (Ultimate 3000 RS Fluorescence Detector, Dionex, Thermo Fisher Scientific, OPA: excitation at 330 nm and emission at 450 nm, FMOC: excitation at 266 nm and emission at 305 nm). Chromeleon v.6.8 software was used to integrate peaks of the derivatives using a reference standard including all amino acids tested.

a

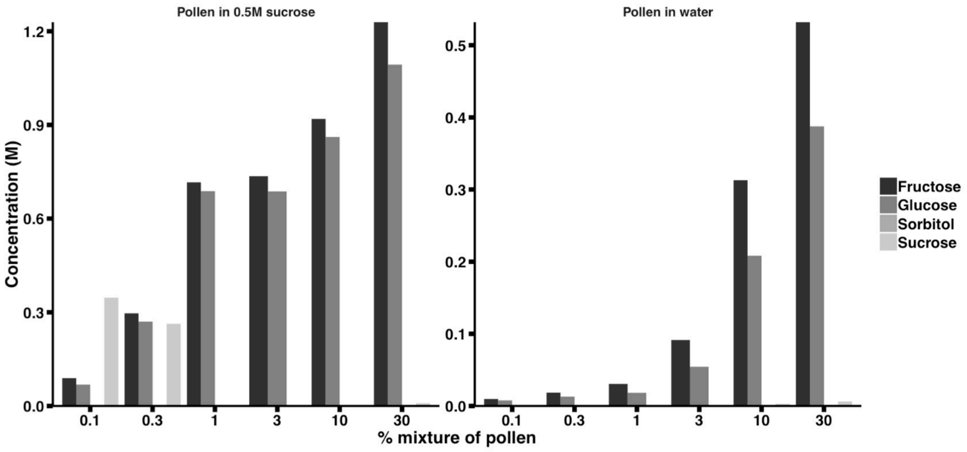

b

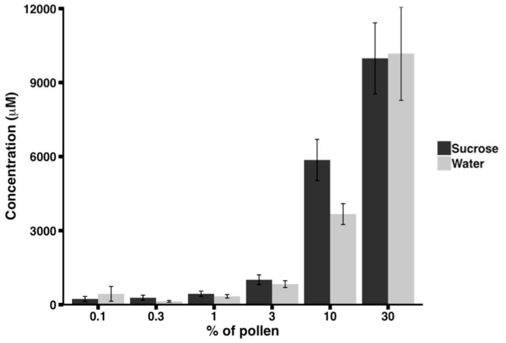

Figure S1: Sugar concentrations in the pollen-sugar and pollen-water solutions.
a) Mainly glucose and fructose were detected in the SPo and WPo solutions at various concentrations. b) Total concentration of aminoacids in SPo and WPo solutions.

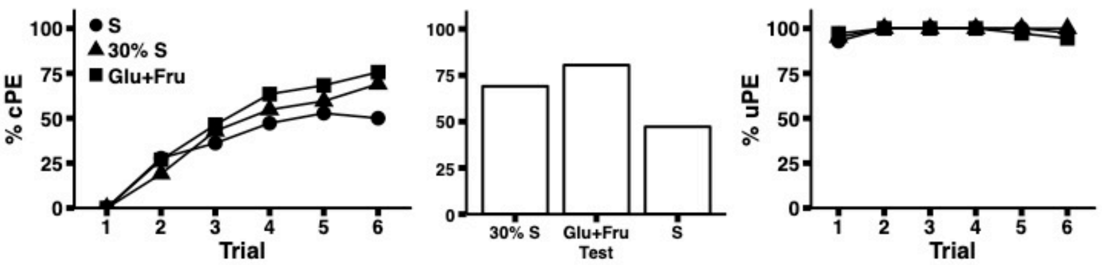

Figure S2: Investigating the effect of sucrose hydrolysis.

To rule out that the adverse effects on acquisition were not simply a result of sucrose hydrolysis, PER conditioning experiments with antennal stimulation were conducted as described above, with bees randomly assigned to one of three treatment groups. *Left panel*: acquisition curves for the CS responses in bees conditioned with either 15% sucrose solution (S, n=36), 30% sucrose solution (n=45) or a mixture containing 1M Glucose and 1M Fructose (n=43). *Middle panel*: CS responses in the final unrewarded test. *Right panel*: Proportion of unconditioned proboscis extensions (uPE) when presenting the US to the antennae during the conditioning trials.

The unconditioned stimulus did not affect the rate and overall level of acquisition (GEE, Treatment X^2_2= 1.51, p = 0.47, Treatment x Trial X^2= 7.58, p = 0.48). Trial number had a significant effect on the level of response across all bees (Trial X^2_4= 56.07, p < 0.001). The unrewarded test revealed small but significant differences between treatment groups (GZLM Test Treatment X_2^2=7.12, p=0.029, LSD S vs. Glu+Fru p=0.005). The uPE responses to the US were very high in all groups across the experiment (GZLM Trial 6 Treatment X_2^2=0.65, p=0.72).

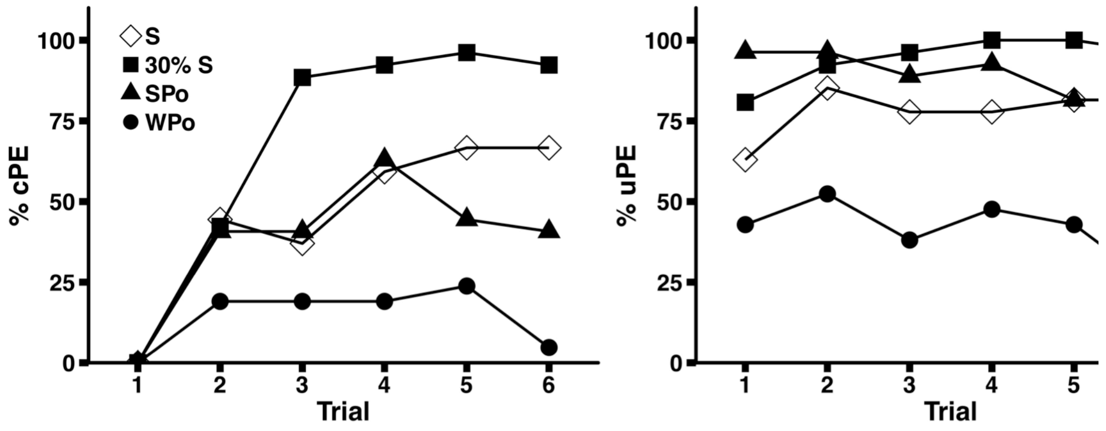

Figure S3: Olfactory conditioning with pollen-supplemented sucrose solution applied to the antennae and proboscis.

Left panel: Acquisition curves in bees rewarded at the antennae and proboscis with sucrose solution (N=27), strong sucrose solution (30% S, N=26), SPo (N=27) or WPo (N= 21). *Right panel*: Unconditioned proboscis extensions (uPE) to the US during each trial.

The type of reward was found to have a significant influence on both the rate and overall level of acquisition (Treatment X^2_3=27.6, Trial X^2_4=20.5, p < 0.001, Treatment x Trial X^2_{12}=33.7, *p*=0.01). Contrast analysis revealed no difference in acquisition between bees reinforced with S and those experiencing SPo (LSD S vs. Spo, *p*=0.411) suggesting that the addition of pollen does not lead to an improvement in learning. In fact, from the fifth trial onwards, bees reinforced with the pollen-sucrose solution show a decline in response to the CS, relative to those receiving the sucrose alone (S). Analysis of the final training trial reveals that the response of bees rewarded with SPo was significantly lower than that of bees rewarded with S (GZLM Trial6 Treatment, X^2_3=22.97, p < 0.001, LSD, *p*=0.048).

Responses to the US varied between groups of bees. On the first trial, a significant effect of treatment was observed (GZLM, Trial 1, Treatment, X^2_3=13.495, *p*=0.004), and interestingly SPo elicited a higher level of response than S (*ca.* 95% compared with 62%, LSD contrast *p*=0.002). By the sixth trial there was no difference in response between bees in both groups (*p*=0.160), and those conditioned with SPo as US were observed to show a slight decline in response to the US over the course of the experiment (from *ca.* 95% to 80%).

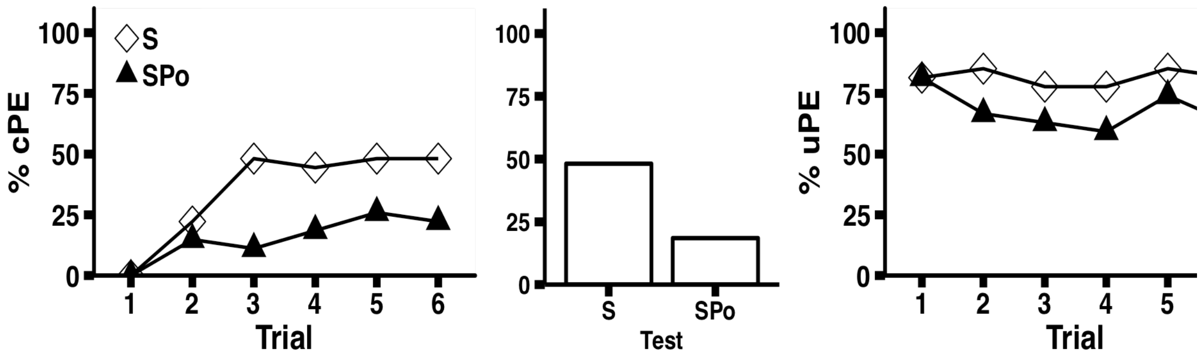

Figure S4: Olfactory PER conditioning performance of partially satiated bees.

Following pre-testing for sensitivity to pollen and sucrose, and one hour prior to the onset of training, individuals were fed 4 µl of 30% sucrose solution (w/w) using a 2 ml capacity Gilmont syringe for precise delivery. This volume is based on the observation by Friedrich et al. (2004) that feeding 15 µl of 1 M sucrose solution four hours prior to conditioning leads to moderate satiation in honeybees. Prior to the start of the experiments bees were randomly allocated to either the group conditioned with 15% sucrose solution (S) or pollen-sucrose mix (SPo). The conditioned stimulus was 1-hexanol (98% purity) diluted in mineral oil to 2.5 M.

Left panel: When bees were moderately satiated prior to the onset of conditioning, the level of acquisition of sucrose-rewarded bees was significantly higher than that of bees conditioned with SPo (Treatment $X^2_1=6.431$, $p=0.011$). *Middle panel*: Proportion of cPE to the CS in an unrewarded test, 10 minutes after the last conditioning trial. Bees conditioned with SPo (N=27) responded at a signficantly lower level than those rewarded with S (N=27, GZLM Test $X^2_1=5.031$, $p=0.025$). *Right panel:* Both groups showed an identical response to the US on the first trial (*ca.* 80%) and though responding on the final trial was not significantly different between groups ($X^2_1=2.237$, $p=0.135$), bees stimulated with the SPo US showed a decline in uPE over the course of training, (from 80 to 62%), whereas bees rewarded with pure sucrose show the same level of responding on the first and last trial, suggesting that SPo bees found the mixture distasteful.

Table S1: Water responses in the taste assays (Experiment 1)

	Taste assay	Factor	d.f.	X^2	p
1	Sucrose alone (control)	**Trial**	**5**	39.44	**<0.001**
		Forager type	1	0.35	0.55
		**Trial x Forager type**	**5**	**12.63**	**0.027**
2	Pollen-sucrose solution	**Trial**	**5**	**28.47**	**<0.001**
		Forager type	1	2.75	0.10
		Trial x Forager type	5	7.47	0.19
3	Proline	**Trial**	**5**	**58.74**	**<0.001**
		Forager type	1	0.23	0.63
		Trial x Forager type	5	5.12	0.40
4	Phenylalanine	**Trial**	**5**	**28.46**	**<0.001**
		Forager type	1	1.67	0.20
		Trial x Forager type	5	5.17	0.27
5	Quinine	**N/A**			
